# BMC Dermatology Reviewer Acknowledgement, 2015

**DOI:** 10.1186/s12895-016-0039-0

**Published:** 2016-02-05

**Authors:** Guangde Tu

**Affiliations:** Room 1504-1505 Financial Plaza, No. 333 Jiujiang Road, Huangpu District Shanghai, 200001 China

## Abstract

The editors of BMC Dermatology would like to thank all our reviewers who have contributed their time to the journal in Volume 15 (2015).

**Rania Abdel Hay**

Egypt

**Ilknur Altunay**

Turkey

**Joao Guilherme Alves**

Brazil

**Christian Apfelbacher**

Germany

**Robert Bransfield**

USA

**Luis Cabero**

Spain

**Barbara Castelnuovo**

Uganda

**Mutlu Cayirli**

Turkey

**Sheau-Chiou Chao**

Taiwan

**Mingyi Chen**

USA

**Chien-Ping Chiang**

Taiwan

**Kyung-Soo Chun**

Republic of Korea

**Giovanni Di Zenzo**

Italy

**Seval Dogruk Kacar**

Turkey

**Jacquelyn Dosal**

USA

**Félix Fanjul-Vélez**

Spain

**Steven Feldman**

USA

**Mari-Ann Flyvholm**

Denmark

**Claire Fuller**

United Kingdom

**Pratik Gahalaut**

India

**Xiaolian Gu**

Sweden

**Carolyn Heckman**

USA

**Elke Heiss**

Austria

**Kedar Inamdar**

USA

**Liselotte Jensen**

USA

**Peter Kaskel**

Germany

**Leon Kircik**

USA

**Lissy Krishnan**

India

**Bhushan Kunar**

India

**Francesco Lacarrubba**

Italy

**Min-Geol Lee**

Republic of Korea

**Jason Lee**

Philippines

**Hauryueh Lee**

Singapore

**Jacob Levitt**

USA

**Marie Loden**

Sweden

**Lois Loescher**

USA

**Gurusamy R. Manoharan**

India

**Amy McMichael**

USA

**Luigi Naldi**

Italy

**Alex Ortega-Loayza**

USA

**Jack S. Resneck**

USA

**Aristóteles Rosmaninho**

Portugal

**Gerhard Schmid-Ott**

Germany

**Marcus Schmitt-Egenolf**

Sweden

**Ali Mohammad Sharifi**

Iran

**Johanna Stoevesandt**

Germany

**Michael Sweeney**

USA

**Yayoi Tada**

Japan

**Wayne Tam**

USA

**Martin Tichy**

Czech Republic

**Jane Tomimori**

Brazil

**Marit B. Veierød**

Norway

**Hywel Williams**

United Kingdom

**Nabiha Yusuf**

USA

